# Carboxymethyl Cellulose/Zn-Organic Framework Down-Regulates Proliferation and Up-Regulates Apoptosis and DNA Damage in Colon and Lung Cancer Cell Lines

**DOI:** 10.3390/polym14102015

**Published:** 2022-05-15

**Authors:** Amr Negm, Mohamed Gouda, Hairul-Islam M. Ibrahim

**Affiliations:** 1 Department of Chemistry, College of Science, King Faisal University, Al-Ahsa 31982, Saudi Arabia; 2 Biochemistry Division, Chemistry Department, Faculty of Science, Mansoura University, Mansoura 35516, Egypt; 3 Biological Science Department, College of Science, King Faisal University, Al-Ahsa 31982, Saudi Arabia; himohamed@kfu.edu.sa

**Keywords:** cellulose nanocomposites, metal–organic framework, biological active agents, biopolymer

## Abstract

A solvothermal technique was used to prepare a Zn–benzenetricarboxylic acid (Zn@BTC) organic framework covered with a carboxymethyl cellulose (CMC/Zn@BTC). Fourier transform infrared spectroscopy (FTIR), field emission scanning electron microscope (FESEM), and Brunauer, Emmett, and Teller (BET) surface area were applied to characterize CMC/Zn@BTC. Moreover, the anticancer, anti-migrative, anti-invasive, and anti-proliferative action of CMC/Zn@BTC nanoparticles were assessed on cancer cell lines. Apoptotic markers and DNA damage were assessed to explore the cellular and biological changes induced by CMC/Zn@BTC nanoparticles. The microscopic observation revealed that CMC controls the surface morphology and surface characteristics of the Zn@BTC. The obtained BET data revealed that the Zn@BTC nanocomposite surface area lowers from 1061 m^2^/g to 740 m^2^/g, and the pore volume decreases from 0.50 cm^3^/g to 0.37 cm^3^/g when CMC is applied to Zn@BTC nanocomposites. The cellular growth of DLD1 and A549 was suppressed by CMC/Zn@BTC, with IC50 values of 19.1 and 23.1 μg/mL, respectively. P53 expression was upregulated, and Bcl-2 expression was downregulated by CMC/Zn@BTC, which promoted the apoptotic process. Furthermore, CMC/Zn@BTC caused DNA damage in both cancer cell lines with diverse impact, 66 percent (A549) and 20 percent (DLD1) compared to cisplatin’s 52 percent reduction. CMC/Zn@BTC has anti-invasive properties and significantly reduced cellular migration. Moreover, CMC/Zn@BTC aims key proteins associated with metastasis, proliferation and programmed cellular death.

## 1. Introduction

Cellulose is a typical biopolymer that is produced at a pace of over 1.5 × 10^12^ tons per year [1]. It is a D-glucose monomer-based natural polymer [1,2,3,4]. It may be extracted from a variety of resources, including plants, wood, algae, tunicate, and bacteria. Cellulose has a wide range of uses [5] e.g., in water treatment [6,7,8], metal adsorption [9], UV radiation shielding [10,11], heat resistance [12], wound healing [13], self-cleaning textiles [14], membranes for antifouling [15], and paper for food-safe usage [16]. It has been used in supercapacitors [17], electronics [18], malleable devices [19], fuel cells [20], and thermal heat-proofing [21]. Drug delivery [22], antifouling [23] and antibacterial uses [24], wound healingand skin tissue engineering [25], vascular grafts [26], and cell-based sensors [27] are some of the biomedical applications [28]. Cellulose-based materials have a good biocompatibility [29] and may be easily changed or printed in three dimensions [30]. As a result, they can be coupled with organic and inorganic nanoparticles [31]. Carboxymethyl cellulose (CMC) is a cellulose derivative with varying degrees of substitution. Researchers are interested in CMC because of its unique properties and growing demand in a variety of fields, such as the food industry, include food packaging, tissue engineering, drug delivery, printing, cosmetics, and dyeing. CMC can be used as an absorbent even at low temperatures because of its strong water interaction and water holding capacity (WHC) [32]. CMC is a water-soluble, hydrophilic, and biodegradable polymer that is utilized for sustainable packaging [33]. Moreover, CMC is utilized to transport drugs e.g., doxorubicin with a high drug-loading content and encapsulation efficiency. The nanoparticles produced have good stability, as well as better safety and tolerability, optimized biodistribution, reduced systemic toxicity, and improved antitumor activity, which indicates a useful role in cancer chemotherapy [34].

Nevertheless, porous hybrid materials-based metal–organic frameworks (MOFs) are made up of organic linkers and metal [35]. MOFs may be made in a variety of ways, including environmentally friendly processes [36]. MOF porosity may be adjusted using a variety of techniques, including ex situ and in situ operations. Different methodologies may be used to create MOFs with hierarchical porous architectures [37,38,39]. Water remediation [40], adsorption [41], pollutant removal [42], catalysis [43], biocatalysis [44], nanomedicine [45], biosensors [46], energy source creation [47], fuel cells [48], and environmental-based applications [49] have all used MOFs. MOFs have environmentally friendly uses [50] and great performance with adjustable features [51]. MOF-based hybrid materials have been reported with other materials; for instance, nanoparticles [52], polymers [53], and covalent organic frameworks [54]. When compared to pure MOFs, MOF hybrid materials exhibit various benefits [55]. Membranes [56], gels [57], and monolithic [57] hybrid materials are all possible. MOFs have also been conjugated with biopolymers [58], polysaccharide [59], carbohydrates [60], and calcium alginate [61], among other biomolecules. Numerous studies focused on the latest developments in MOFs in biomedical sectors, including disease diagnosis and drug delivery for cancer therapy, bioimaging, antibacterial applications, biosensing, and biocatalysis [62]. Their small particle sizes enable them to penetrate cells and exert their cellular biochemical function [63]. The benefits of in-cooperation materials, for instance excellent biocompatibility, easy production, vast surface area, and broad application, were merged in biomolecules–MOFs hybrid materials.

The rising prevalence of cancer has prompted researchers to look for safe and effective chemotherapy treatments. Chemotherapeutic drugs are now the most effective way to treat cancer by inducing necrosis and/or apoptosis [64,65,66]. Novel multitarget anticancer drugs that affect many molecular sites to promote cancer cell death are in great demand. Furthermore, coating MOFs with a polymer is an attractive strategy for overcoming their drawbacks, for instance, weak stability and low biocompatibility. The purpose of this research was to apply a hydrothermal method for preparing a carboxymethyl cellulose (CMC)-containing Zn@BTC mesoporous nanomaterial (CMC/Zn@BTC). Moreover, the assessment of anti-proliferative, DNA damage induction, and modulation of apoptotic key genes (p53 and Bcl-2) and the anticancer effectiveness of the produced CMC/Zn@BTC nanocomposites.

## 2. Materials and Methods

### 2.1. Materials

Carboxymethyl cellulose with a degree of substitution of 1.0 with average Mt. 18000 was purchased from (Merck-Sigma, Darmstadt, Germany). Absolute ethanol 99%, zinc nitrate hexahydrate (Zn (NO_3_)_2_·6H_2_O) 99.8%, 1,3,5-benzenetricarboxylic acid 98.99%, and the rest of the supplies were obtained from (Merck-Sigma, Darmstadt, Germany).

### 2.2. Synthesis of (CMC/Zn@BTC)

The solvothermal technique was used to prepare the CMC/Zn@BTC nanocomposite, with accordance to reported methods [67]. A total of 5.2 g of Zinc nitrate hexahydrate, 1.0 g BTC, and 0.33 g CMC were combined in 30 mL water: ethanol (50:50) (*v*:*v*) solution in a typical reaction. The combination was solvothermal treated for 25 h at 120 °C. Lastly, the solid sample was rinsed with ethanol, and CMC/Zn@BTC was produced by drying at 75 °C using a vacuum and transferred in a desiccator. The same process was used to synthesize pure Zn@BTC without the use of CMC.

### 2.3. Characterization

SEM-JOELF was used to evaluate the surface morphology of CMC, Zn@BTC, and CMC/Zn@BTC nanocomposite (Tokyo, Japan). In a scanning electron microscope (SEM), a low-energy electron beam is sent to the sample and scans the surface of the sample. When the beam enters the material, many interactions occur, resulting in the emission of photons and electrons from or near the surface of the sample. The samples are gold-coated with a thickness of 40–60 nm and then analyzed under a microscope. For the SEM image analysis, a 15.0 kV accelerating voltage was applied. A FTIR-8400S Spectrometer (SHIMADZU, Kyoto, Japan) was used to determine FTIR spectra which are collected in the range of 400 to 4000 cm^−1^. The crystal structures of the CMC, Zn@BTC, and CMC/Zn@BTC nanocomposite was determined by XRD (Rigaku, Tokyo, Japan) using Cu K radiation (= 0.154 nm) at 40 kV and 30 mA with a 2 range of 5 to 80°.

### 2.4. Specific Surface Area

A programmed gas sorption analyzer was used to compute N_2_ adsorption–desorption (Ji Nan RunZhi Technology, Shanghai, China). The produced materials were outgassed for 4–6 h before being analyzed for N_2_ adsorption–desorption isotherms at 77.35 K using a volumetric approach that reflects additional adsorption isotherms [68]. The Brunauer–Emmett–Teller (BET) approach was used to calculate the specific surface area of the prepared samples based on isotherm.

### 2.5. Biological Activity Evaluation

#### 2.5.1. Cell Culture and MTT Assay

To test the cytotoxicity of CMC/Zn@BTC, colon (DLD1) and lung (A549) cancer cell lines were incubated in a DMEM medium containing appropriate antibiotics, and incubated for 48 h [69]. After the incubation time, the wells were filled with dilutions of CMC/Zn@BTC (10–100 µM/mL) in triplicate, and the incubation period was continued for another 48 h. The active viable cells were monitored via their ability to convert MTT into a purple color. After that, 20 µL MTT was added, the plates were stirred, and the supernatant liquid was removed. Next, 100 mL DMSO was poured into each well. After 15 min, optical density was recorded at 540 nm. 

#### 2.5.2. Invasion Assay

A Matrigel-coated Boyden chamber was used to performed this assay [70]. Cells were grown in culture medium with CMC/Zn@BTC in the upper chamber. The chambers were left overnight at 37 °C. Next, 750 µL DMEM containing 10% FBS were added to the lower chamber. Plates were kept for 36 hrs at 37 °C. Then, the medium was removed, and cells were fixed by 3.7% buffered formalin for few minutes. Cells were permeabilized by absolute methanol for 20 min. The invasive cells were stained with giemsa stain for 15 min. Stain was aspirated from chambers and washed twice with phosphate-buffered saline; non-invasive cells were cleaned off using cotton swabs before they were counted.

#### 2.5.3. Migration Assay

Migration assay was performed using a scratch wound-healing method [71]. A total of 2 mL of diluted cells were added to each well of a six-well plate containing 175,000 cells per milliliter. Cell cultures were left to adhere overnight. Normal serum media were exchanged with a low serum medium. After that, using sterile tips, the wells were scratched to form a straight line at the center of the confluent cells well. Then, the wells were incubated with CMC/Zn@BTC or DMSO for 24 h, and the migration was monitored.

#### 2.5.4. DNA Damage Assessment

DNA damage assessment was applied to inspect the DNA damage induced by CMC/Zn@BTC, as previously mentioned [72,73]. Cells were mixed with various dilutions of CMC/Zn@BTC and incubated for 24 h. Lysis solution was mixed with cells and left for 2 h at 55 °C. The DNA pellet was mixed with TE buffer containing RNaseA until it dissolved. DNA damage was assessed using agarose gel electrophoresis technique. Ethidium bromide was used to stain the gel, which was then photographed using a transilluminator.

#### 2.5.5. RT-qPCR

Total RNA was extracted according to Li, H. et al. [74]. cDNA was synthesized from RNA using reverse transcriptase (TaKaRa Biotechnology, Otsu, Japan). P53 and BCL-2 transcript expression levels were determined using the Applied Biosystems Real-Time PCR system. qPCR was performed in a thermocycler (Applied Biosystems, Foster, CA, USA) with a reaction volume of 10 μL containing 0.03 μg complementary DNA product, 2 μM forward and reverse primers, and the SYBR^®^ FAST qPCR reagent (Kapa Biosystems, Wilmington, MA, USA). Primer’s sequences was as follows: BCL-2 FW “5′-TTGGCCCCCGTTGCTT-3′” and RV “5′-CGGTTATCGTACCCCGTTCTC-3′”; P53 “F-5′-GTATTTCACCCTCAAGATCC-3′” and “R-5′-TGGGCATCCTTTAACTCTA-3′”; and GAPDH “F-5′-GAGAAACCTGCCAAGTATG-3′”, and “R-5′-GGAGTTGCTGTTGAAGTC-3′”. The procedure included 40 cycles of 95 °C for 5 s and 60 °C for 34 s. As a reference control, GAPDH was used. The relative expression of each gene was calculated using the comparative 2Ct technique [75].

## 3. Results and Discussion

### 3.1. FT-IR

**Figure 1:**
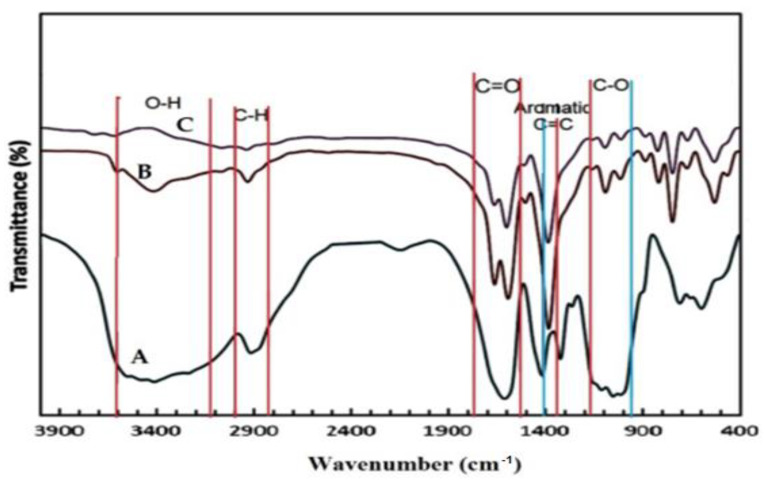
FT-IR spectra of (A) CMC; (B) Zn@BTC and (C) CMC/Zn@BTC.

Figure 1 shows FT-IR spectra of pure CMC, Zn@BTC, and CMC/Zn@BTC. The carboxylate groups in Zn@BTC and CMC were stretched symmetrically and asymmetrically at 1508, 1725 cm^−1^ and 1539, 1401 cm^−1^, correspondingly. The benzene basic vibration was recorded at 1630 cm^−1^ for Zn@BTC. The out-of-plane vibrations of BTC carboxylate groups for Zn@BTC are connected to the many bands seen in the 1250–600 cm^−1^ range. In comparison to Zn@BTC, all discovered distinctive peaks in CMC/Zn@BTC with increasing intensity show that the structure of Zn@BTC and CMC was intact. The FT-IR data might provide solid indication for connections among the –COOH of CMC and BTC through Zn^2+^ of CMC/Zn@BTC.

### 3.2. XRD

**Figure 2:**
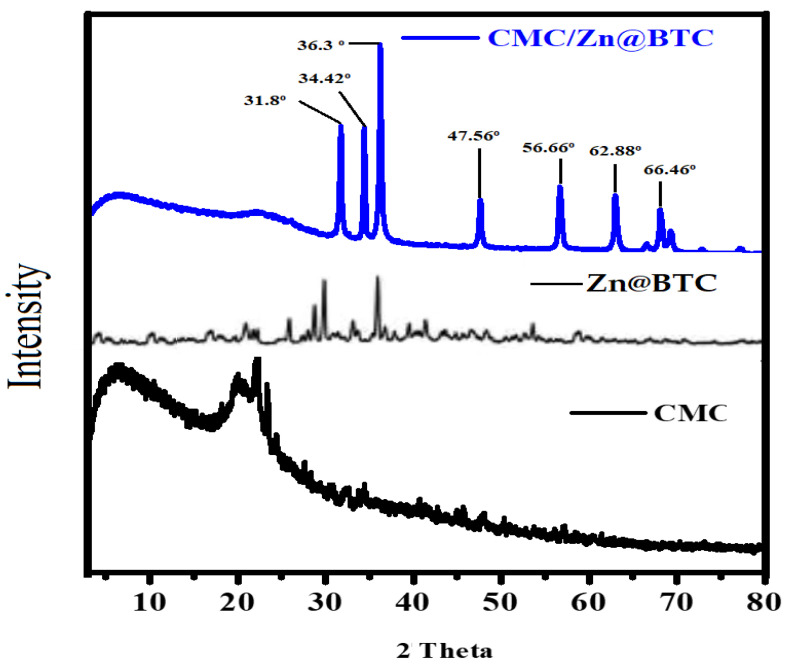
XRD pattern of CMC, Zn@BTC, and CMC/Zn@BTC.

XRD patterns of CMC, Zn@BTC, and CMC/Zn@BTC are shown in Figure 2. The amorphous character of CMC is connected to the wide replication at a 2θ significance of around 20°. A border peaks at 2θ values equal 31.8°, 34.42°, 36.3°, 47.56°, 56.66°, 62.88°, and 66.46°, which is matched with (100, 002, 101, 102, 110, 103, and 200) of the JCPDS data and revealed that the produced ingredients were ZnO nanoparticles with zincite phase. Figure 2 shows the distinctive peaks for Zn@BTC at 31.8° and 36.3°, which also showed in the pattern for CMC/Zn@BTC when the Zn-BTC was coated on the CMC surface. These findings show that the Zn@BTC crystal structure in the composite product is substantially retained. According to the XRD data, the crystalline structure of the Zn@BTC was not affected after mixing with CMC in the CMC/Zn@BTC and suggested that Zn@BTC powder was present at the CMC surface, as illustrated in Figure 2. It was discovered that Zn@BTC was firmly lodged in the CMC lattice and that the nanoparticles did not dislodge from the CMC surface following washing with ethanol. According to the XRD data, the composition of Zn@BTC and CMC in the CMC/Zn@BTC nanocomposite did not affect the crystalline structure of the Zn@BTC component. Using the Scherrer equation and the diffraction intensity of the (101) peak, the typical particle size of the prepared nanocomposites was calculated according to Scherrer Equation (1) to calculate the mean particle size (D) based on the XRD line increase measurement:

D = 0.89λ/(βCosθ)(1)

where λ is the wavelength, β is the ZnO (101) line’s full width at half maximum (FWHM), and θ is the diffraction angle. The presence of a distinct line widening of the diffraction peaks indicates that the produced materials are nanometer-sized with an average diameter of 30 nm.

### 3.3. SEM Examination

**Figure 3:**
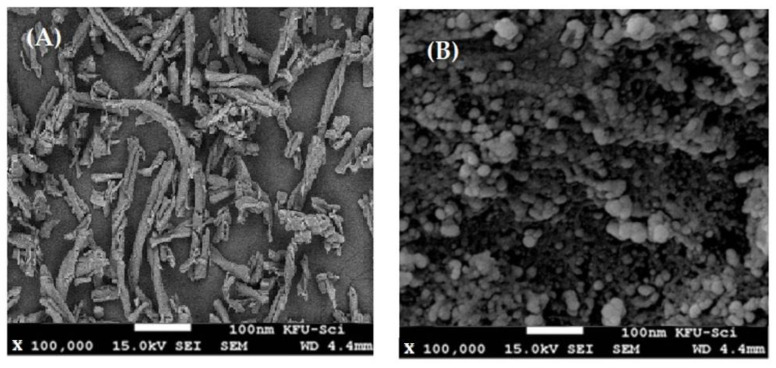
SEM of (**A**) Zn@BTC and (**B**) CMC/Zn@BTC.

Morphologies of CMC and CMC/Zn@BTC nanocomposite were examined using SEM. The CMC/Zn@BTC nanocomposite has a well-defined morphology (Figure 1B). The standardized spread of Zn@BTC on CMC formed a necklet structure. It also created a substantial amount with a large surface area and a particle diameter of around 30 nm. Furthermore, Figure 3B shows the spongy structure of CMC/Zn@BTC on CMC with square and lamellar forms.

### 3.4. EDX Analysis

**Figure 4:**
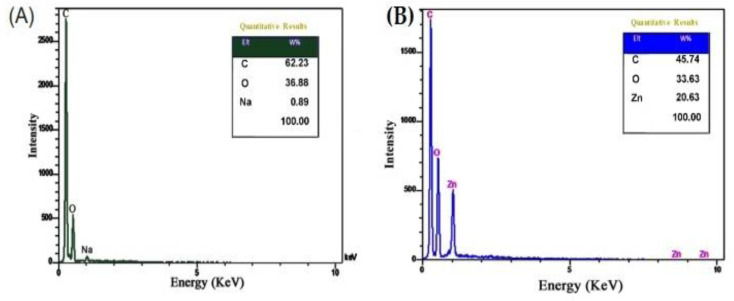
EDX analysis of (**A**) CMC, and (**B**) CMC/Zn@BTC.

Figure 4 shows the collected element information of prepared samples using EDX analysis. The presence of C, O, and Na is indicated by signals in the CMC spectrum (Figure 4A) with weight percentages of 62.23, 36.68, and 0.89 W%, respectively. As shown in Figure 4B, the CMC/Zn@BTC EDX spectrum revealed the presence of just C, O, and Zn with 45.74%, 33.63%, and 20.63% weight percent, respectively, indicating a successful formation with excellent purity. When comparing CMC/Zn@BTC to pure CMC, the presence of Zn was found.

### 3.5. Surface Area Investigation

**Figure 5:**
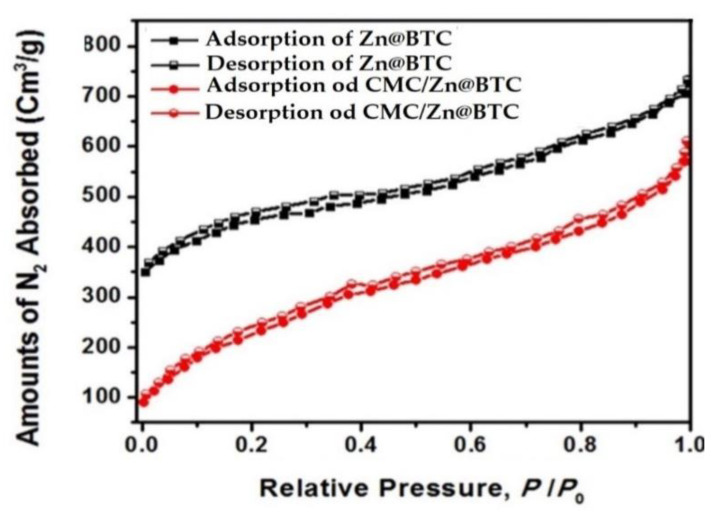
N_2_ adsorption–desorption isotherms of Zn@BTC and CMC/Zn@BTC.

N_2_ adsorption–desorption isotherms of Zn@BTC and CMC/Zn@BTC nanocomposite are shown in Figure 5. N_2_ adsorption diminishes at low relative pressure in CMC/Zn@BTC because CMC is primarily confined to the micropores of Zn@BTC rather than mesopores [76]. Zn@BTC has a microporous structure with a diameter of less than 2 nm, and aggregation of micropores can generate mesopores with a diameter of 2 to 50 nm. Grainy positions may occur inside the pore structure because of Zn@BTC agglomeration, as seen in the SEM data. The development of mesoporous results in N_2_ adsorption at agglomerated grainy spots. The approach of Horvath–Kawazoe that deals with the numerical assessment of gas adsorption in distinct opening pores was used to calculate the nanocomposite’s pore volume. Adsorption at pores, according to this estimate, happens only at a given relative pressure, which is determined by the energy of interaction between adsorptive molecules and adsorbent [77]. Total surface area (BET) and empirical scattering of pore width (D) at several comparative pressures of N_2_ adsorption at 77 K are used to calculate the pore volume (V).

V = D × BET/4(2)

when CMC is applied to Zn@BTC, the nanocomposite surface area was decreased from 1061 m^2^/g to 740 m^2^/g, and the pore volume was lowered from 0.50 cm^3^/g to 0.37 cm^3^/g.

### 3.6. Biological Activity

#### 3.6.1. Anti-Proliferative Activity

**Figure 6:**
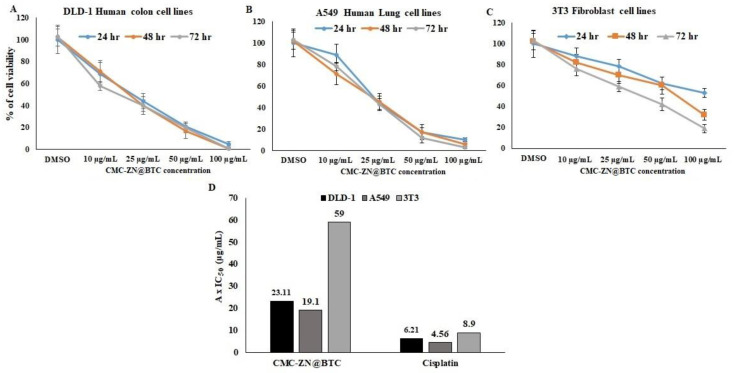
In vitro anticancer activity of CMC/Zn@BTC on (**A**) colon cancer cell lines, (**B**) lungcancer cell lines, (**C**) normal cell lines. (**D**) represents collective graph to compare against cisplatin.

The MTT assay is a type of viability assay applied to examine target compounds’ cellular effects to acquire more information about the biological processes, which can be altered by potential chemotherapeutic drugs [78]. The MTT test was applied to determine the cytotoxic effect of CMC/Zn@BTC. The amount of surviving cancer cells was diminished after exposure to CMC/Zn@BTC for 24, 48, and 72 h, with its most noteworthy effect towards colon cancer cell lines. Earlier research has found that cellulose derivative-based nanoparticles exhibit a significantly higher effectiveness than cellulose itself [79]. The MTT test revealed the anti-proliferative action of CMC/Zn@BTC (Figure 6); it reduced the growth of A549 and DLD1 cell lines and exhibited IC_50_ values of 23.1 and 19.1 µg/mL for the colon and lung cancer cell lines, respectively. The values of IC50 of Zn@BTC against DLD1, A549 and 3T3 were 104.1 L, 161.4 and 183.3, respectively. This toxicity was significantly lower when compared to CMC/ZN@BTC-treated cell lines. This means that the CMC composite form increased the cytotoxicity of Zn@BTC, which may be because CMC increased the bioavailability of the compound, as it already did with previous drugs e.g., doxorubicin.

#### 3.6.2. Migration and Invasion

**Figure 7:**
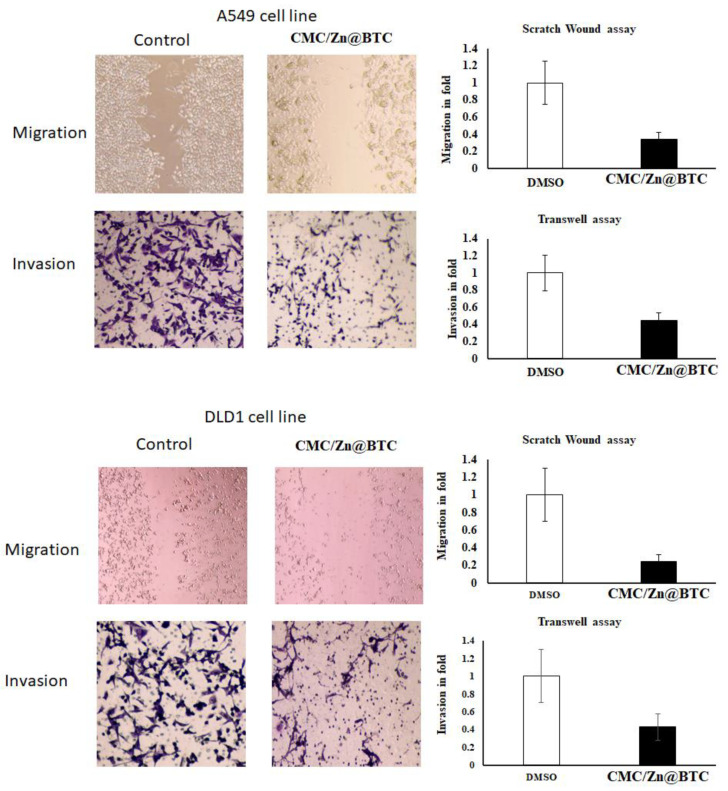
Anti-invasive and -migrative effects of CMC/Zn@BTC on cancer cell lines.

Apart from apoptosis, metastasis is an important key player in cancer progression. Migration, adhesion, and invasion are integral process associated with metastasis [80]. Matrigel drop invasion assay is a well-known experiment that is used to assess in vitro cancer cell invasion and migration. The purpose of this experiment was to see if CMC/Zn@BTC influences the motility of human cancer cells. Treatment of cancer cell lines with CMC/Zn@BTC (IC50) decreased migration and invasion, and migration of treated cells was slower than the control group. Upon CMC/Zn@BTC treatment, the wound healing time was delayed, and the cell motility was inhibited (Figure 7). This was validated by the potential migrative marker BCL2, using quantitative real-time PCR.

The effect of CMC/Zn@BTC on cell invasion was explored by applying the Boyden chamber assay. Upon CMC/Zn@BTC treatment, the number of invasive DLD1 and A549 cells decreased by 65%. The restriction of cellular invasion and migration supports the potential anticancer efficacy of CMC/Zn@BTC, which would resist cancer cells’ proliferation [81]. CMC/Zn@BTC showed anti-migration and anti-invasion activity, which might maximize its effectiveness as an anti-metastatic agent in cancer treatment.

#### 3.6.3. DNA Damage Activity

**Figure 8:**
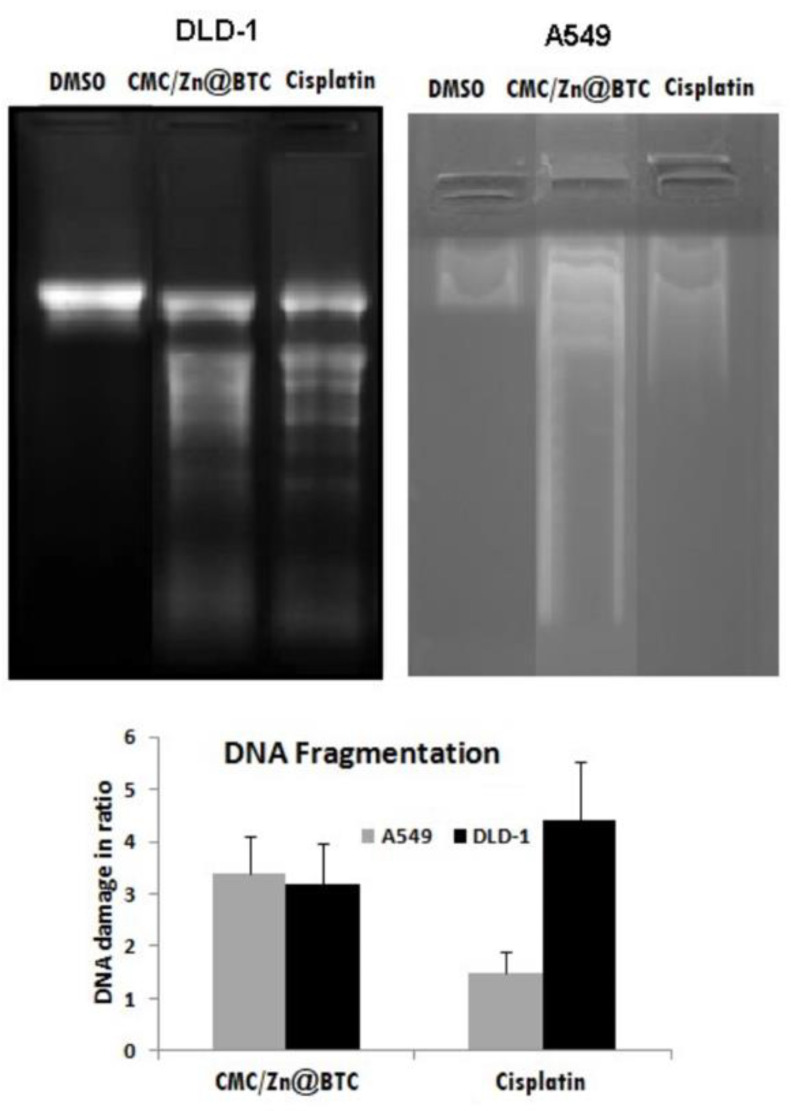
DNA damage effect of CMC/Zn@BTC.

The DNA damage effect of CMC/Zn@BTC was assessed and compared to cisplatin. Upon treatment with CMC/Zn@BTC, the DNA-laddering pattern was recorded using a DNA damage test. CMC/Zn@BTC caused a marked incline in DNA damage and induced more fragmented laddering pattern in cancer cell lines compared to cisplatin (Figure 8). The DNA fragmentation induced by CMC/Zn@BTC is one of the pathways which leads to potent apoptotic induction, which is a hallmark of cellular death [82]. Thus, CMC/Zn@BTC can trigger nuclear DNA fragmentation into nucleosomal units and pave the way to subsequent cell death, mimicking the action of caspase-activated DNase that induce chromosomal DNA fragmentation leading to apoptosis [83]. The DNA damage evidenced that ZN@BTC did not induce DNA damage and apoptosis at 100 ug/mL concentration, whereas DLD1 was slightly more sensitive than the A549 and 3T3 cell lines. This means that the CMC composite form increased the DNA fragmentation ability of Zn@BTC.

#### 3.6.4. qRT-PCR of p53 and BCL-2 Expression

**Figure 9:**
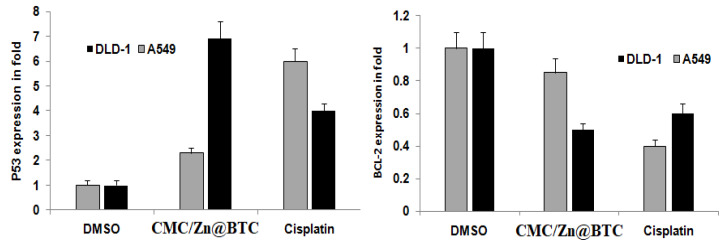
Effect of CMC/Zn@BTC on the expression of P53 and BCl-2 in cancer cell lines.

Apoptosis is a type of controlled cellular death which maintains a good survival/death ratio in tissues. Apoptosis deficiency is apparent in cancer cells, whereas elevated apoptosis can trigger degenerative illnesses. Apoptotic signals contribute to the maintenance of genomic integrity. Restricting apoptosis stimulates cancer. As a result, apoptosis modulation is a successful therapeutic strategy for cancer [84]. The impact of CMC/Zn@BTC on important apoptotic genes’ expression; p53 and Bcl-2, was investigated. CMC/Zn@BTC exhibited dose-dependent up-regulation of p53 and down-regulation of BCL-2 (Figure 9). The p53 gene is crucial for the induction of programmed cell death as well as the preservation of genomic integrity [85]. CMC/Zn@BTC exhibited a 70 percent and 15 percent increase in p53 expression in colon and lung cancer cell lines, respectively, compared to a 40 percent and 60 percent reduction in cisplatin-treated cells. CMC/Zn@BTC enhanced declines in Bcl-2 expression 54 percent and 18 percent in colon and lung cancer cell lines, respectively, compared to 55 percent and 60 percent declines with cisplatin. Increased p53 expression increases chemotherapeutic sensitivity in cancer cells [86], whereas BCL-2 inhibits apoptosis [87] Thus, CMC/Zn@BTC markedly increased p53 expression while decreasing BCL-2. These findings potentially strengthen their anti-proliferative efficacy in the treatment of cancer.

## 4. Conclusions

A hydrothermal approach was used to effectively produce mesoporous nanomaterials of Zn at 1,3,5-benzentricarboxylic acid framework integrated into carboxymethyl cellulose. The surface morphology of synthesized Zn@BTC is exceedingly smooth, as demonstrated by SEM pictures, and no morphological alterations were seen. Furthermore, particle diameters ranged from 22 to 30 nm. The crystal structure of Zn@BTC was not distorted by the combination of Zn@BTC and CMC in the CMC/Zn@BTC nanocomposite, and the structure of Zn@BTC was well preserved, according to the XRD data. CMC/Zn@BTC nanocomposite treatment reduced cell proliferation and enhanced DNA damage. Furthermore, it caused apoptosis and decreased metastatic efficacy in cancer cell lines. Anti-proliferation, apoptosis, and DNA damage induction suggest that CMC/Zn@BTC could be used as an anti-proliferative agent for cancer cells to retard cancer growth and development.

## Data Availability

The authors confirm that the data of this study are available within the article. Raw data are available from the corresponding author upon request.
